# Methotrexate promotes the release of granulocyte–macrophage colony-stimulating factor from rheumatoid arthritis fibroblast-like synoviocytes via autocrine interleukin-1 signaling

**DOI:** 10.1186/s13075-024-03406-6

**Published:** 2024-10-11

**Authors:** Beatrice Bergström, Tilia Selldén, Miriam Bollmann, Mattias N. D. Svensson, Anna-Karin Hultgård Ekwall

**Affiliations:** 1 Department of Rheumatology and Inflammation Research, Institute of Medicine, Sahlgrenska Academy, University of Gothenburg, Gothenburg, Sweden; 2 SciLifeLab, University of Gothenburg, Gothenburg, Sweden; 3 Department of Rheumatology, Division 3, Sahlgrenska University Hospital, Gothenburg, Sweden

**Keywords:** Rheumatoid arthritis, Synovitis, Fibroblast-like synoviocyte, Anti-rheumatic drugs, Cytokines

## Abstract

**Background:**

Activated fibroblast-like synoviocytes (FLS) are drivers of synovitis and structural joint damage in rheumatoid arthritis (RA). Despite the use of disease-modifying drugs, only about 50% of RA patients reach remission in real-world settings. We used an unbiased approach to investigate the effects of standard-of-care methotrexate (MTX) and a Janus kinase inhibitor, tofacitinib (TOFA), on gene expression in RA-FLS, in order to identify untargeted disease mediators.

**Methods:**

Primary RA-FLS were activated by stimulation with interleukin-1β (IL-1β) or platelet-derived growth factor + IL-1β in the presence or absence of MTX or TOFA, with or without additional inhibitors. Co-cultures of synovial cells were performed in direct and indirect systems. Cells were collected for RNA sequencing or qPCR, and supernatants were analyzed for protein concentrations.

**Results:**

Six thousand three hundred fifty genes were differentially expressed, the majority being upregulated, in MTX-treated activated RA-FLS and 970 genes, the majority being downregulated, in TOFA-treated samples. Pathway analysis showed that MTX had largest effects on ‘Molecular mechanisms of cancer’ and TOFA on ‘Interferon signaling’. Targeted analysis of disease-associated genes revealed that MTX increased the expression of cell cycle-regulating genes but also of pro-inflammatory mediators like IL-1α *(IL1A*) and granulocyte–macrophage colony-stimulating factor, GM-CSF (*CSF2*)*.* The MTX-promoted expression of *CSF2* in activated RA-FLS peaked at 48 h, could be mediated via either NF-κB or AP-1 transcription factors, and was abrogated by IL-1 inhibitors (IRAK4 inhibitor and anakinra). In a co-culture setting, MTX-treatment of activated RA-FLS induced *IL1B* expression in macrophages.

**Conclusions:**

MTX treatment induces secretion of IL-1 from activated RA-FLS which by autocrine signaling augments their release of GM-CSF. This unexpected effect of MTX might contribute to the persistence of synovitis.

**Supplementary Information:**

The online version contains supplementary material available at 10.1186/s13075-024-03406-6.

## Background

Rheumatoid arthritis (RA) is a chronic systemic autoimmune disease that predominantly affects the joints, but is also associated with substantial comorbidities such as cardiovascular disease and osteoporosis [1]. Only about 50% of patients achieve clinical remission in real-world settings despite the recent decades of improvements using biologic and synthetic disease modifying anti-rheumatic drugs (DMARDs) [2, 3]. This painful fact demonstrates the knowledge gap regarding molecular disease mechanisms as well as potential off-target drug effects, and emphasizes the need for novel treatment strategies.

Accumulated evidence highlights the critical role of fibroblast-like synoviocytes (FLS) in the pathogenesis of RA [4–6]. Activated stromal cells and infiltrating immune cells trigger and amplify signals leading to persistent inflammation and joint damage. Sustained activation of synovial effector cells in the RA joint is created by secretion of interleukin (IL)-1β and tumour-necrosis factor (TNF) by macrophages, triggering release of granulocyte–macrophage colony-stimulating factor (GM-CSF) and IL-6 from FLS which in turn activates macrophages [7, 8]. Platelet-derived growth factor (PDGF) is also produced in high amounts in the RA joint and potentiates the effects of cytokines such as IL-1 on FLS [9]. Plasma levels of PDGF were reported to be increased in a cohort of active RA patients compared to healthy controls and correlated with CRP [10], further highlighting its significance in disease pathogenesis.

Initiation of treatment with DMARDs early in the RA disease course is key to abrogate inflammation and prevent structural damage and disability [11]. Low-dose methotrexate (MTX) is still the first-line therapy for RA. The many postulated anti-inflammatory effects of MTX include e.g. release of adenosine, leading to reduced cytokine production [12], and inhibition of dihydrofolate reductase (DHFR), leading to activation of the transcription factor AP-1 and normalized proliferation rate of immune cells, in particular T-cells [13, 14]. Also, MTX was shown to suppress Janus kinase (JAK)/Signal transducer and activator of transcription (STAT) signaling in a macrophage cell line, although not as profoundly as compared to a JAK inhibitor [15]. Effects of MTX on other effector cell types in RA, such as stromal cells, have been less investigated. One study reported MTX-mediated inhibition of nuclear factor-κB (NF-κB) activity in RA-FLS [16] and we demonstrated normalized levels of cell cycle-regulating genes, including the RA-associated risk gene *LBH*, by MTX in these cells [17].

Still, 50–60% of RA patients respond inadequately to MTX in monotherapy and require additional drugs [18–20]. JAK inhibitors, targeting the intracellular signaling of multiple cytokines and a few hormones and growth factors [21], are the newest class of drugs in RA treatment and have demonstrated higher efficacy compared to MTX [22]. The JAK inhibitor tofacitinib has been reported to inhibit TNF-induced, type I interferon-mediated, chemokine expression in RA-FLS [23, 24], but other effects on these cells remain incompletely characterized.

In this study, we performed an unbiased investigation of the effects of methotrexate and tofacitinib on activated RA-FLS. The aim was to identify pathogenic responses from these cells that are not resolved by treatment and thus may perpetuate or even worsen the inflammation.

## Methods

### Patients

Human synovial tissue specimens were obtained from patients with RA undergoing arthroplasty or synovial biopsy at Sahlgrenska University Hospital in Sweden. Anti-rheumatic treatment, including MTX, had been stopped two weeks before surgery.

Blood samples were collected from a longitudinal cohort of early RA patients [25]. Analysis was performed on serum from 24 subjects at diagnosis (before initiation of prednisone or DMARDs) and after two years of anti-rheumatic treatment. Demographic and clinical data were collected (joint assessment, patient and physician global assessments of disease activity, erythrocyte sedimentation rate and C-reactive protein, radiographs of hands and feet, autoantibodies, and therapy), and characteristics of these patients are summarized in Supplementary Table 1.

All RA patients fulfilled the American College of Rheumatology 2010 revised criteria for the disease [26]. The procedures and study protocol were approved by the Regional Ethics Committee of Gothenburg and the Swedish Ethical Review Authority, respectively. All patients gave written informed consent.

### Cell culture

Primary FLS lines (*n* = 9 different FLS lines were used for this study) were established from synovial tissue as described earlier [27] and cultured in Dulbecco’s modified Eagle’s medium (DMEM) GlutaMAX supplemented with antibiotics (penicillin/streptomycin, gentamicin) and 10% heat-inactivated fetal bovine serum (FBS) (all from Gibco, Carlsbad, CA, USA), in a humidified 5% CO_2_ and 37 °C atmosphere. Cells in passages 4–8 were used in experiments.

### Stimulations and treatments

Primary RA-FLS were seeded in 12–24-well plates, serum-starved overnight in DMEM with 1% FBS and then activated with the recombinant human proteins PDGF-BB (20 ng/mL; Invitrogen, Carlsbad, CA, USA), IL-1β (2 ng/mL; R&D Systems, Minneapolis, MN, USA), or IL-1α (2 ng/mL; BioLegend, San Diego, CA, USA), at concentrations based on previous work [17]. For studies of treatment effects, cells were pre-treated for 24 h with MTX (1 µM; Metoject, Medac GmbH, Wedel, Germany) or tofacitinib (2.5 µM; CP-690,550, Selleck Chemicals, Houston, TX, USA) prior to stimulation with IL-1β or PDGF + IL-1β in fresh 1% FBS medium with or without the drug for the indicated time (Supplementary Fig. 1). After 24–72 h, cells and supernatants were collected for analysis. There were no visible signs of cell death in the samples at the time of harvest. Each experiment was repeated ≥ 3 times with similar results. The selected concentrations of MTX and tofacitinib were based on earlier in vitro dose–response studies [17] and were in the range of plasma concentrations achieved by dosages given for RA [28].

Inhibition experiments were performed with the following compounds (with references supporting the concentrations used): NF-κB inhibitor (0.5 µM [29]; BAY 11–7085, MedChemExpress (MCE), NJ, USA), Jun N-terminal kinase (JNK) inhibitor (0.5 µM [30]; JNK-IN-8, MCE), IL-1 receptor associated kinase 4 (IRAK4) inhibitor zimlovisertib (1 µM [31]; PF-06650833, MCE), or IL-1 receptor antagonist anakinra (500 ng/mL [32]; Kineret, Sobi, Stockholm, Sweden).

### Ex vivo synovial tissue bioassay

An ex vivo bioassay was performed according to Kuo et al*.* [33]. Briefly, RA synovial tissue was enzymatically dissociated with 50 µg/mL Liberase TM and 100 µg/mL deoxyribonuclease I (both from Roche, Basel, Switzerland) and filtered through a 70 µm cell strainer. Cells were resuspended in 10% FBS-DMEM, seeded in a 96-well plate (2 × 10^5^ cells/well) and subjected to treatment with MTX or TOFA for 48 h prior to RNA isolation.

### FLS-macrophage co-culture assay

Monocytes were isolated from buffy coats of healthy blood donors using EasySep Human Monocyte Isolation Kit (STEMCELL Technologies, Vancouver, BC, Canada), and differentiated into macrophages by incubation for one week in presence of 50 ng/mL macrophage colony-stimulating factor (M-CSF; PeproTech, Cranbury, NJ, USA) [34]. The macrophages were then detached and seeded onto a transwell insert that was placed in a 24-well plate with activated/treated RA-FLS. After 48 h of co-culture, in 1% FBS-DMEM, RNA was isolated separately from the two cell types.

### RNA isolation and qPCR

Total RNA was isolated from cells using RNeasy Micro Kit (Qiagen, Hilden, Germany) according to the manufacturer’s protocol and quantified with a NanoDrop One spectrophotometer (Thermo Scientific, Waltham, MA, USA)*.* Complementary DNA was synthesized using High-Capacity cDNA Reverse Transcription Kit (Applied Biosystems, Foster City, CA, USA). Quantitative real-time PCR (qPCR) was performed on a ViiA 7 Real-Time PCR system, using pre-designed TaqMan primer–probe sets (Supplementary Table 2) from Applied Biosystems. Cycle threshold (Ct) values were normalized to glyceraldehyde 3-phosphate dehydrogenase (GAPDH) as reference gene and fold change in mRNA expression was calculated using the delta-delta Ct method.

### RNA sequencing

The experimental design is described in Supplementary Fig. 1. Libraries were prepared using TruSeq Stranded Total RNA Sample Preparation Kit with Ribo-Zero Gold (Illumina, San Diego, CA, USA). RNA sequencing (RNA-seq) was performed on an Illumina NextSeq 500 system with paired-end 75-bp read length. After data quality assessment using FastQC and filtering using PRINSEQ, reads were mapped to the human reference genome (hg19, UCSC assembly, Feb 2009) with STAR and quantified by HTSeq.

### Immunoassay

Levels of GM-CSF in cell culture supernatants were measured using Bio-Plex Pro Human Cytokine GM-CSF Set, and levels of cytokines in patient serum samples were measured using Bio-Plex Pro Human Cytokine Screening Panel 48-plex (both purchased from Bio-Rad Laboratories, Hercules, CA, USA), according to the manufacturer’s instructions. Data were acquired on a Bio-Plex 200 system and concentrations were calculated by the Bio-Plex Manager software (Bio-Rad).

### Statistical and bioinformatic analysis

Data are presented as mean ± standard error of the mean (SEM) or median as indicated. After normality testing (Shapiro–Wilk test), statistical significance was evaluated by paired Student’s *t* test, one-way repeated measures ANOVA followed by post-hoc test for multiple comparisons, or Wilcoxon signed-rank test, where appropriate. Statistical analysis of differences in gene expression by qPCR was performed on delta Ct values. Data were analyzed with GraphPad Prism version 10 (GraphPad Software, La Jolla, CA, USA). *p* < 0.05 was considered significant.

For RNA-seq data, differential gene expression between treated and untreated samples were analyzed using DESeq2 with Wald test and Benjamini–Hochberg *p*-value adjustment for multiple testing [35]. Genes were considered differentially expressed if adjusted *p* < 0.05. The differentially expressed genes were subject to Ingenuity Pathway Analysis (IPA, Qiagen) or Gene Set Enrichment Analysis [36] (GSEA pre-ranked with 1000 permutations for selected gene sets available in MSigDB: NFKAPPAB_01 and AP1_01, http://www.broad.mit.edu/gsea) as indicated.

## Results

### MTX induces broad transcriptional upregulation in activated RA-FLS

We have previously reported that MTX promotes gene expression of the cell cycle regulators *CDKN1A* (cyclin-dependent kinase inhibitor 1A, p21), *TP53* (tumor protein p53) and *LBH* (Limb-bud and heart development) in activated (PDGF + IL-1β-stimulated) RA-FLS, with most pronounced effects after 48 h [17]. In search of novel MTX-regulated genes in these cells, we performed RNA-seq on samples subjected to either 1) PDGF + IL-1β (untreated activated control), 2) PDGF + IL-1β + MTX, or 3) PDGF + IL-1β + tofacitinib (TOFA) as a comparator drug at this time point.

**Fig. 1:**
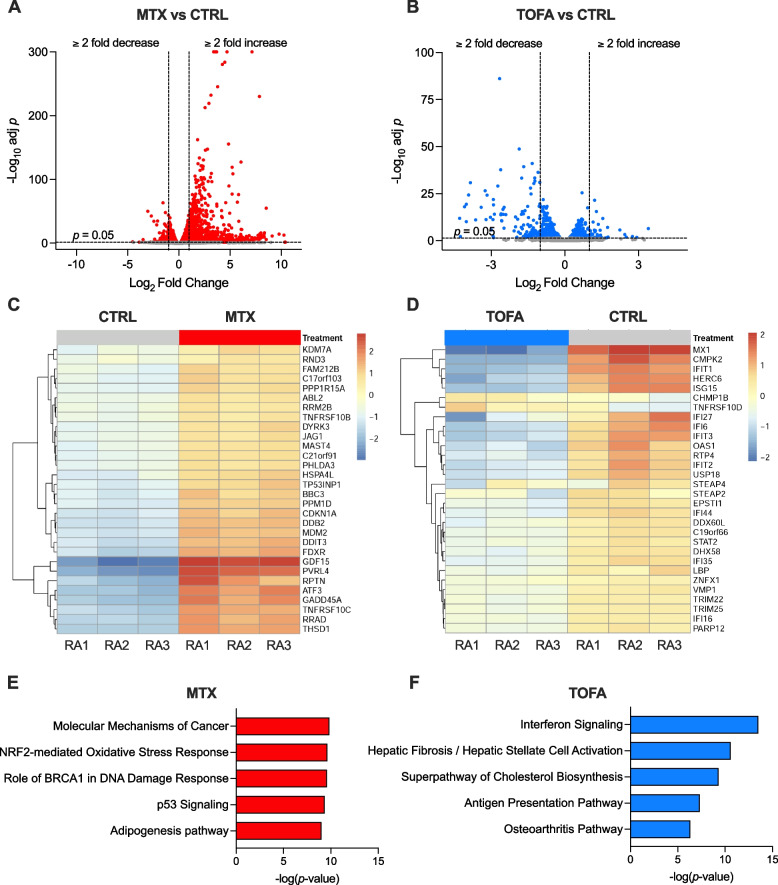
Transcriptomic changes in PDGF + IL-1β-activated RA-FLS treated with methotrexate (MTX) or tofacitinib (TOFA). Primary RA-FLS in passage 5 were pre-treated with drug or vehicle for 24 h followed by stimulation with PDGF-BB + IL-1β in the presence or absence of the drug for 48 h prior to RNA-seq. **A**-**B** Volcano plots showing differentially expressed genes (DEGs; adjusted *p* < 0.05) for MTX (**A**) and TOFA (**B**) versus untreated activated control (CTRL). Dashed lines at adjusted *p* = 0.05 and at fold change =  ± 2. **C-D** Heatmaps with hierarchical clustering of the top 30 DEGs for MTX (**C**) and TOFA (**D**). **E**–**F** Top five canonical pathways identified by Ingenuity Pathway Analysis of the DEGs for MTX (**E**) and TOFA (**F**)

A total of 6,350 genes were differentially expressed (DEGs; adj. *p* < 0.05) in MTX-treated activated RA-FLS samples compared to untreated activated control (Fig. 1A). Out of these, 1,253 genes were increased ≥ twofold and 154 genes were decreased ≥ twofold. In contrast, at 48 h, TOFA treatment resulted in 970 DEGs out of which only 36 genes were increased ≥ twofold and 118 genes were decreased ≥ twofold (Fig. 1B). The top 30 DEGs for each treatment are shown in Fig. 1C and D, respectively. Pathway analysis of all DEGs demonstrated that MTX had largest effects on ‘Molecular mechanisms of cancer’ together with other pathways related to cell cycle regulation (Fig. 1E), strengthening our previous findings [17]. For TOFA, on the other hand, the most significant pathway identified by IPA was ‘Interferon signaling’ (Fig. 1F). This is also consistent with previous reports of TOFA-mediated inhibition of type I interferon signaling in cytokine-stimulated RA-FLS [23].

### MTX targets disease-associated genes in RA-FLS

**Fig. 2:**
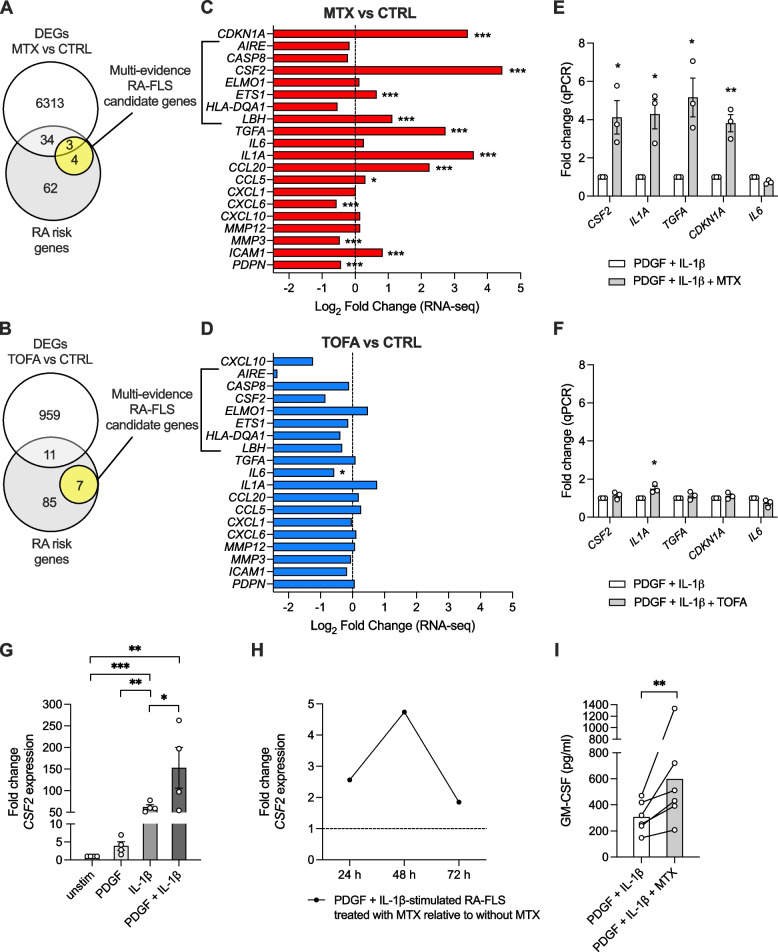
Effects of methotrexate (MTX) and tofacitinib (TOFA) on RA-associated risk genes in PDGF + IL-1β-activated RA-FLS. **A**-**B** Venn diagrams of differentially expressed genes (DEGs) by MTX (**A**) and TOFA (**B**), RA risk genes (identified by genome-wide association studies) and multi-evidence RA-FLS candidate genes (identified by integrative omics analysis [39]). **C**-**D** RNA-seq-based fold changes of pathogenic RA-FLS genes in response to MTX (**C**) or TOFA (**D**). **E**–**F** Validation of RNA-seq data by qPCR of activated RA-FLS treated with MTX (**E**) or TOFA (**F**). **G*** CSF2* gene expression by qPCR of RA-FLS stimulated with PDGF-BB and/or IL-1β for 24 h. **H** Time course of *CSF2* expression in PDGF + IL-1β-activated, MTX-treated RA-FLS. **I** GM-CSF protein expression in cell culture supernatants of activated RA-FLS treated with or without MTX for 48 h. Bar graphs show mean ± SEM and circles represent individual values. *p < 0.05, **p < 0.01, ***p < 0.001 by Wald test (**C**, **D**), paired *t* test (**E**, **F**, **I**) or one-way repeated measures ANOVA with multiple comparisons (**G**)

In a previous study, we found that around 25% of genome-wide association study-identified RA risk genes [37] were differentially expressed in TNF + IL-1β-stimulated RA-FLS [38]. Here, we found that 36% (37/103) of the RA risk genes were differentially expressed in the MTX-treated activated RA-FLS compared to untreated activated control (Fig. 2A; Supplementary Table 3), while only 11% (11/103) were differentially expressed by TOFA (Fig. 2B; Supplementary Table 4).

We further assessed the effects of the drugs on genes known to be of relevance for RA-FLS pathogenicity, in particular a set of seven “multi-evidence” candidate genes identified by integrative analysis of RA risk genes together with transcriptomic and epigenomic data of RA as compared to OA and normal FLS [39]. The targeted analysis also included other genes associated with an activated, pathogenic RA-FLS phenotype [4, 38], such as cytokines, chemokines, matrix-degrading enzymes, and adhesion molecules. As a confirmation of earlier report, MTX increased the expression of the cell cycle-regulating genes *CDKN1A* and *LBH* [17] (Fig. 2C), the latter also being one of the multi-evidence RA-FLS candidate genes. Two more multi-evidence genes were differentially expressed by MTX, namely *CSF2* and *ETS1*. Surprisingly, MTX *increased* the gene expression of *CSF2* (encoding GM-CSF) and also other pro-inflammatory mediators like *IL1A* (IL-1α), *CCL20* (macrophage inflammatory protein-3α) as well as *TGFA* (transforming growth factor α). Other RA-FLS genes with relevance for the RA pathogenesis, like *MMP3* (matrix metallopeptidase 3) and *PDPN* (podoplanin), were instead decreased by MTX treatment. TOFA reduced the gene expression of *CXCL10* (interferon-γ-inducible protein) as expected, although the effect was not statistically significant at 48 h (Fig. 2D). For a majority of the investigated genes, including the multi-evidence RA-FLS candidate genes, there were no significant differences in response to TOFA treatment at this time point. qPCR confirmed upregulation of *CSF2* (4.1 ± 0.8 fold versus untreated activated control, *p* = 0.022), *IL1A* (4.3 ± 0.8 fold, *p* = 0.02), *TGFA* (5.2 ± 1.1 fold, *p* = 0.015) and *CDKN1A* (3.8 ± 0.4 fold, *p* = 0.008) in RA-FLS by MTX (Fig. 2E). TOFA had minor effects on these genes as measured by qPCR (Fig. 2F).

### MTX enhances the release of GM-CSF from activated RA-FLS

As mentioned, *CSF2* is a candidate gene for pathogenic features of RA-FLS [39, 40] and also a top-90 gene upregulated in RA-FLS after TNF + IL-1β stimulation [38]. In an experimental set-up without drug treatment, we confirmed that *CSF2* is induced by IL-1β stimulation of RA-FLS (62 ± 5.6 fold, *p* < 0.0001) compared to unstimulated control (Fig. 2G). PDGF was also able to induce *CSF2* to some extent (3.9 ± 1.2 fold compared to unstimulated), but the combination of PDGF + IL-1β resulted in a striking synergistic increase in *CSF2* expression (150 ± 47 fold change compared to unstimulated, *p* = 0.0026). However, the synergistic effect was not observed for all RA-FLS samples, demonstrating the biological variance, different response kinetics (e.g. due to passage number) or priming of primary cell cultures [41].

In the presence of MTX, the *CSF2* expression of PDGF + IL-1β-activated RA-FLS was augmented with a peak at 48 h (Fig. 2H). Furthermore, increased production of GM-CSF at the protein level could be detected already at 48 h in the supernatants of these cells in response to MTX treatment (Fig. 2I).

### MTX promotes inflammatory crosstalk between FLS and macrophages

**Fig. 3:**
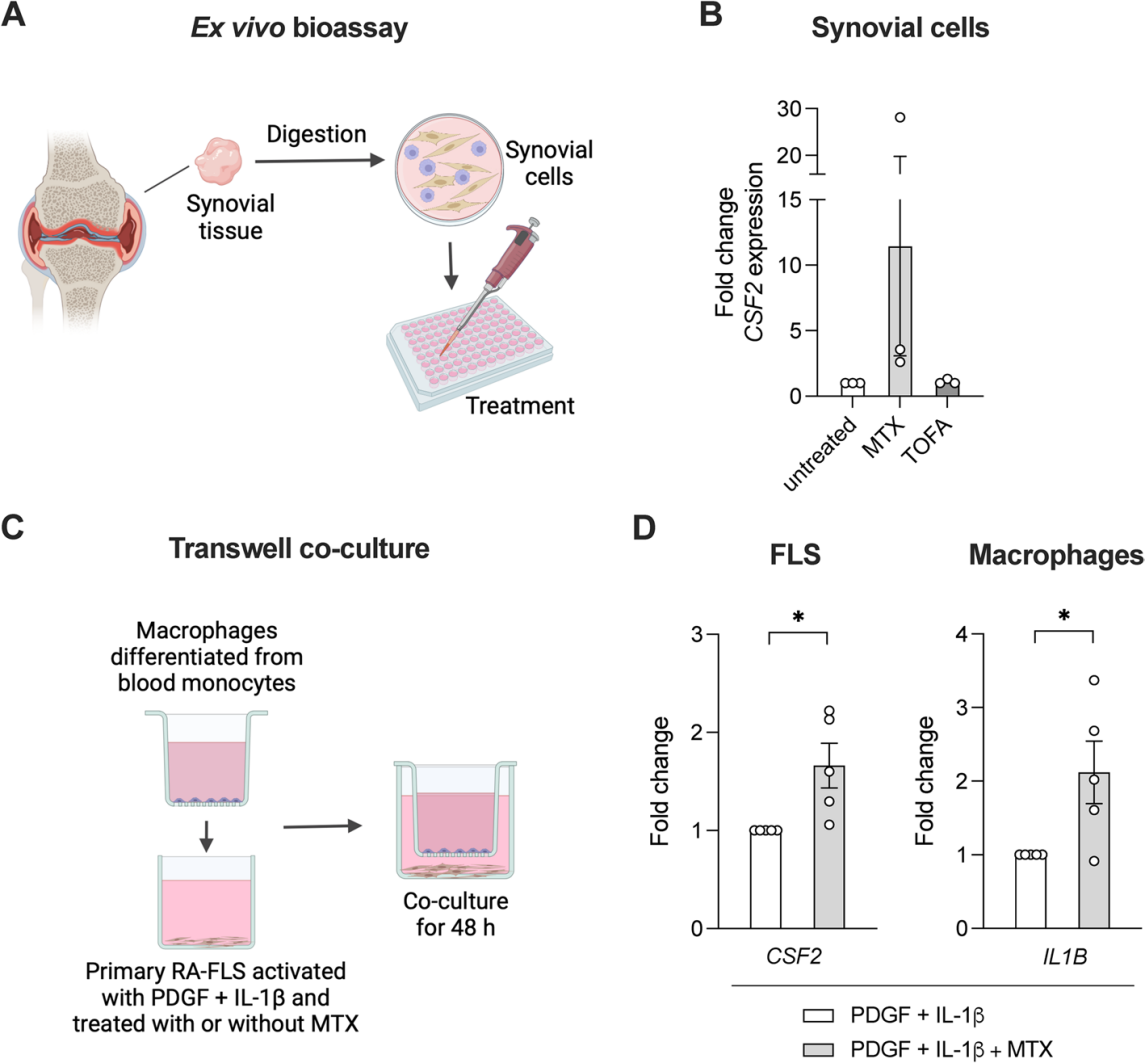
Effects of anti-rheumatic treatments on FLS-macrophage crosstalk in co-culture. **A** In an ex vivo bioassay, cells were dissociated from RA synovial biopsies, plated and subjected to treatment with MTX or TOFA for 48 h. **B** Gene expression of *CSF2* by qPCR of the synovial cells. **C** For an indirect co-culture assay, monocytes were isolated from blood and differentiated into macrophages by M-CSF, then seeded on a transwell insert. Primary RA-FLS were pre-treated with MTX or vehicle for 24 h, then activated with PDGF + IL-1β in the presence or absence of MTX for 24 h, before addition of the top insert with macrophages. **D** Following co-culture for 48 h, qPCR analysis was performed on FLS (*CSF2*) and macrophages (*IL1B*) separately. Illustrations created with BioRender.com. *p < 0.05 by paired *t* test

To study the effects of DMARD-treated RA-FLS on other synovial cell types, we first performed an ex vivo synovial bioassay [33]. Cells were dissociated from RA synovial tissue and cultured with MTX or TOFA for 48 h (Fig. 3A). In this mixed-cell population, gene expression of *CSF2* was again increased by MTX but not by TOFA (Fig. 3B).

To further investigate the interaction between different synovial cell types, and in particular effects of GM-CSF secretion, a transwell system was set up where macrophages were indirectly co-cultured with activated and treated RA-FLS (Fig. 3C). qPCR analysis of the RA-FLS compartment confirmed an upregulated *CSF2* expression in response to MTX (1.7 ± 0.2 fold versus untreated activated control, *p* = 0.03) (Fig. 3D). Macrophages co-cultured with MTX-treated activated RA-FLS demonstrated higher gene expression of *IL1B* compared to macrophages co-cultured with untreated activated RA-FLS (2.1 ± 0.4 fold, *p* = 0.04) (Fig. 3D). Similar results were observed when macrophages were exposed to supernatants (after washout) from MTX-treated activated RA-FLS (Supplementary Fig. 2).

### MTX promotes GM-CSF expression in RA-FLS via increased IL-1 signaling

To explore mechanisms underlying the augmented secretion of GM-CSF by activated RA-FLS in the presence of MTX, inhibition of different pathways was evaluated.

**Fig. 4:**
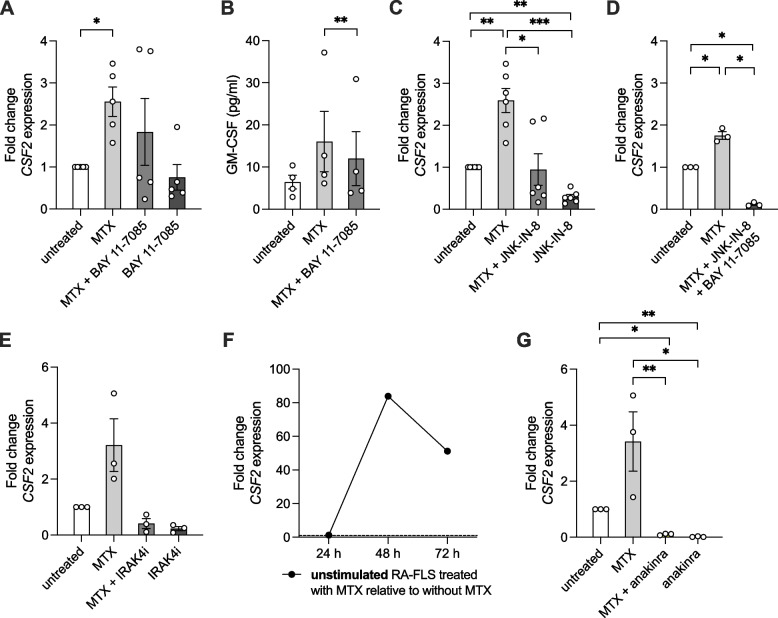
Effects of different inhibitors on MTX-promoted *CSF2*/GM-CSF expression in activated RA-FLS. After pre-treatment, RA-FLS were activated with IL-1β for 12 h (except in Fig. 4F) and then treated with or without MTX and/or inhibitor for 24 h. We tested the effects of **A**-**B** NF-κB inhibitor (BAY 11–7085) (*CSF2* gene expression by qPCR (**A**) and GM-CSF protein levels in cell culture supernatants (**B**)), **C** JNK inhibitor (JNK-IN-8), **D** the combination of BAY 11–7085 and JNK-IN-8, or **E** IRAK4 inhibitor (IRAK4i, zimlovisertib). **F** Time course of *CSF2* expression in unstimulated MTX-treated RA-FLS. **G** Effects of IL-1 receptor antagonist anakinra on MTX-promoted *CSF2* expression in activated RA-FLS. Bar graphs show mean ± SEM and circles represent individual values. *p < 0.05, **p < 0.01, ***p < 0.001 by one-way repeated measures ANOVA with multiple comparisons

First, we investigated the roles of the transcription factors NF-κB and AP-1 which are both involved in inflammatory gene expression, including regulation of *CSF2* at least in immune cells [42–44]. Moreover, MTX has previously been reported to modulate NF-κB activation in RA-FLS [16]. Another documented effect of MTX is inhibition of DHFR, leading to increased reactive oxygen species production which in turn activates Jun N-terminal kinases (JNKs) [13] and subsequently the transcription factor AP-1 in some cell types. We found an enrichment both of genes targeted by NF-κB (e.g. *IL1A*, *CCL5*, *CXCL6*, *ICAM1*) and genes targeted by AP-1 (e.g. *CDKN1A*, *IL6*), among the genes upregulated by MTX compared to untreated activated control in our RNA-seq dataset (GSEA normalized enrichment score 1.35, FDR = 0.018, and 1.74, FDR < 0.001, respectively) (Supplementary Fig. 3A-B). When IL-1β-activated RA-FLS were subjected to a combination of MTX and the NF-κB activation inhibitor BAY 11–7085, the MTX-mediated increase in *CSF2* expression was abrogated in some, but not all, of the tested RA-FLS (fold change to untreated activated control 1.8 ± 0.8 with MTX + BAY 11–7085 versus 2.6 ± 0.3 with MTX alone, *p* = 0.52) (Fig. 4A). This effect was confirmed at the protein level (Fig. 4B). Similarly, a JNK inhibitor (JNK-IN-8) could also suppress the augmented *CSF2* expression in response to MTX (fold change to untreated activated control 0.9 ± 0.4 with MTX + JNK-IN-8 versus 2.6 ± 0.3 with MTX alone, *p* = 0.048) (Fig. 4C). However, as with BAY 11–7085, the extent of the inhibitory response to JNK-IN-8 varied largely between RA-FLS derived from different patients. When inhibiting both of these transcription factors simultaneously, MTX could not promote the expression of *CSF2* in any of the tested RA-FLS lines (Fig. 4D).

To understand whether MTX acts directly on enzymes in the IL-1 pathway in the cytoplasm or via indirect extracellular mediators, we first tested a selective IL-1 receptor-associated kinase 4 inhibitor (IRAK4i), zimlovisertib, in the experimental setting. Zimlovisertib has previously been demonstrated to reduce cytokine and matrix metalloproteinase release from stimulated RA-FLS [45]. Addition of IRAK4i reduced the MTX-mediated increase in *CSF2* expression in activated RA-FLS (fold change to untreated activated control 0.4 ± 0.2 with MTX + IRAK4i versus 3.2 ± 0.9 with MTX alone, *p* = 0.054) (Fig. 4E), indicating that MTX acts early on the IL-1 pathway upstream of this kinase. Considering the upregulation of *IL1A* by MTX in the RNA-seq data, we speculated that MTX increases *CSF2* expression via autocrine IL-1α signaling. IL-1α binds to the same IL-1 receptor as IL-1β and has a similar capacity to induce *CSF2* in RA-FLS (Supplementary Fig. 4). Indeed, even in *unstimulated* RA-FLS, i.e. without prior IL-1β activation, MTX could also increase the expression of *CSF2* with a peak at 48 h (Fig. 4F). Further supporting the theory of IL-1α secretion as a mechanism for the MTX-promoted increase in *CSF2*, the addition of the recombinant IL-1 receptor antagonist anakinra could efficiently abrogate this effect (Fig. 4G).

### Synovial expression of *CSF2, IL1B and IL1A* correlates with disease activity

Earlier studies have shown elevated levels of GM-CSF and IL-1β in synovial fluid [46, 47] and synovial membrane [48] in RA compared to other types of inflammatory arthritis or osteoarthritis. There are also reports of higher plasma concentrations of GM-CSF [49] and IL-1β [50, 51] in RA patients compared to healthy controls.

**Fig. 5:**
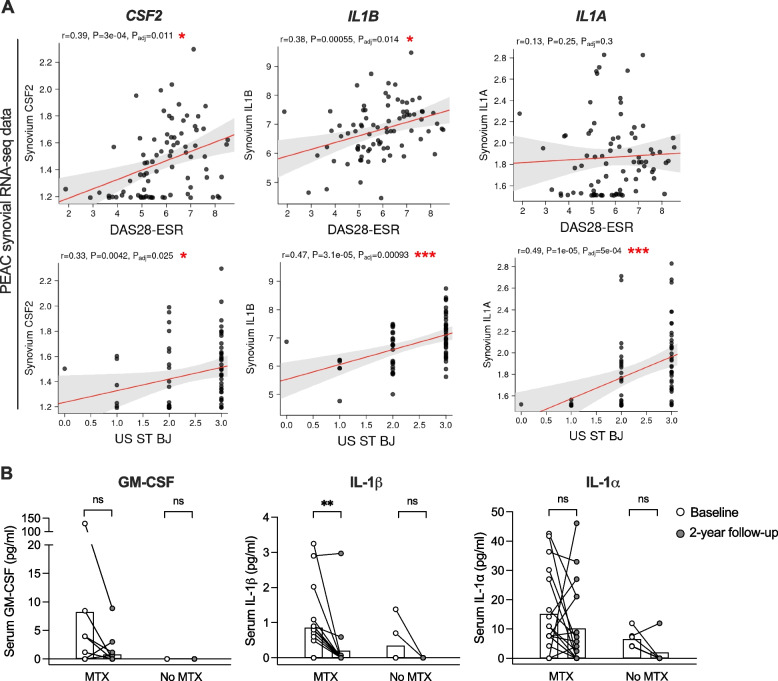
Clinical relevance of GM-CSF, IL-1α and IL-1β in early RA. **A** Data from the PEAC cohort of early untreated RA patients. Correlations between synovium gene expression of *CSF2*, *IL1B* and *IL1A*, respectively, and disease activity score (DAS28-ESR) or ultrasound synovial thickness (US ST BJ). Adjusted *p < 0.05, ***p < 0.001. **B** Serum levels of GM-CSF, IL-1α and IL-1β in another early RA cohort, measured at baseline (diagnosis) and after two years of treatment that included MTX (*n* = 18) or not (*n* = 6). Bar graphs show median and circles represent individual values. **p < 0.01 by Wilcoxon signed-rank test

The PEAC study provides publicly available RNA-seq data of treatment-naïve RA patients with a disease duration of < 12 months and enables comparison of blood or synovium transcripts with different clinical, histological or radiographic parameters (https://peac.hpc.qmul.ac.uk) [52]. In this dataset, the transcript levels of *CSF2*, *IL1B* as well as *IL1A* correlated with ultrasound synovial thickness of the biopsied joint, supporting the significance of these factors in the joint pathology of early RA. Moreover, there was also a positive correlation between both *CSF2* and *IL1B* gene expression in the synovium and Disease Activity Score 28-joint count with Erythrocyte Sedimentation Rate (DAS28-ESR) (Fig. 5A).

Using a cohort of early, untreated RA patients (Supplementary Table 1), we assessed protein concentrations of GM-CSF, IL-1β and IL-1α in blood samples collected at diagnosis and after two years of anti-rheumatic therapy. Treatment was grouped as MTX (monotherapy or combined with other drugs) or no MTX (other DMARDs). Serum levels of GM-CSF and IL-1β were generally low and in many cases undetectable (Fig. 5B). The overall disease remission rate by DAS28-CRP (< 2.6) at follow-up in this cohort was 58%. Interestingly, while the levels of IL-1β were significantly reduced at two years as compared to baseline (Fig. 5B), there was no significant reduction in IL-1α. The levels of IL-1α were unchanged or increased between baseline and two-year follow-up for 33% of the patients in the MTX group, but only 17% in the no MTX group. The blood levels of IL-1α at follow-up did not correlate with disease activity assessed by DAS28-CRP, swollen joint count nor tender joint count (Supplementary Fig. 5). No qualitative assessment of joints with ultrasound was performed in this cohort.

## Discussion

Despite advances in the treatment of RA, a considerable proportion of patients still do not reach sustained remission [18, 53]. There is clearly a need for better understanding of the cellular and molecular drivers of synovial inflammation and how they are affected by treatment. Also, the molecular effects, beneficial or untoward, of conventional DMARDs like methotrexate on synovial cells are far from completely understood. Attention has been drawn to inflammatory stromal cells, like activated FLS, as key players in the RA joint pathology and as potential therapeutic targets. Indeed, in the recent R4RA trial, a fibroblast signature of the synovium was associated with non-response to three biological drugs [54].

Here, we investigated the effects of MTX and TOFA on the transcriptome of activated RA-FLS. We hypothesized that pathogenic functions of RA-FLS are insufficiently targeted by currently used therapies and may continue to drive disease progression. Low-dose MTX has been the gold standard of RA therapy for more than thirty years, but its mechanisms of action on RA-FLS remain to be elucidated. Our RNA-seq data revealed that MTX induces an overall upregulation of gene expression in activated RA-FLS. Unexpectedly, MTX promoted the expression not only of known cell cycle-regulating genes leading to benefits like reduced FLS proliferation, but also induced expression of important RA disease mediators such as *IL1A* and *CSF2*. In a microarray study from 2007, MTX treatment of unstimulated immortalized RA synovial fibroblasts resulted in differential expression of 29 genes, the majority being upregulated, including *IL1A* and *IL1B,* which supports our findings [55]. Moreover, MTX has been reported to induce expression of pro-inflammatory cytokines (IL-1, IL-6) in monocytic cell lines [56]. It has been speculated that such effects could contribute to some toxicities of MTX, like mucositis and pneumonitis. Potentially, persistent untargeted IL-1 signaling in connective tissue and endothelium could also be of significant importance for co-morbidities like cardiovascular disease [57].

**Fig. 6:**
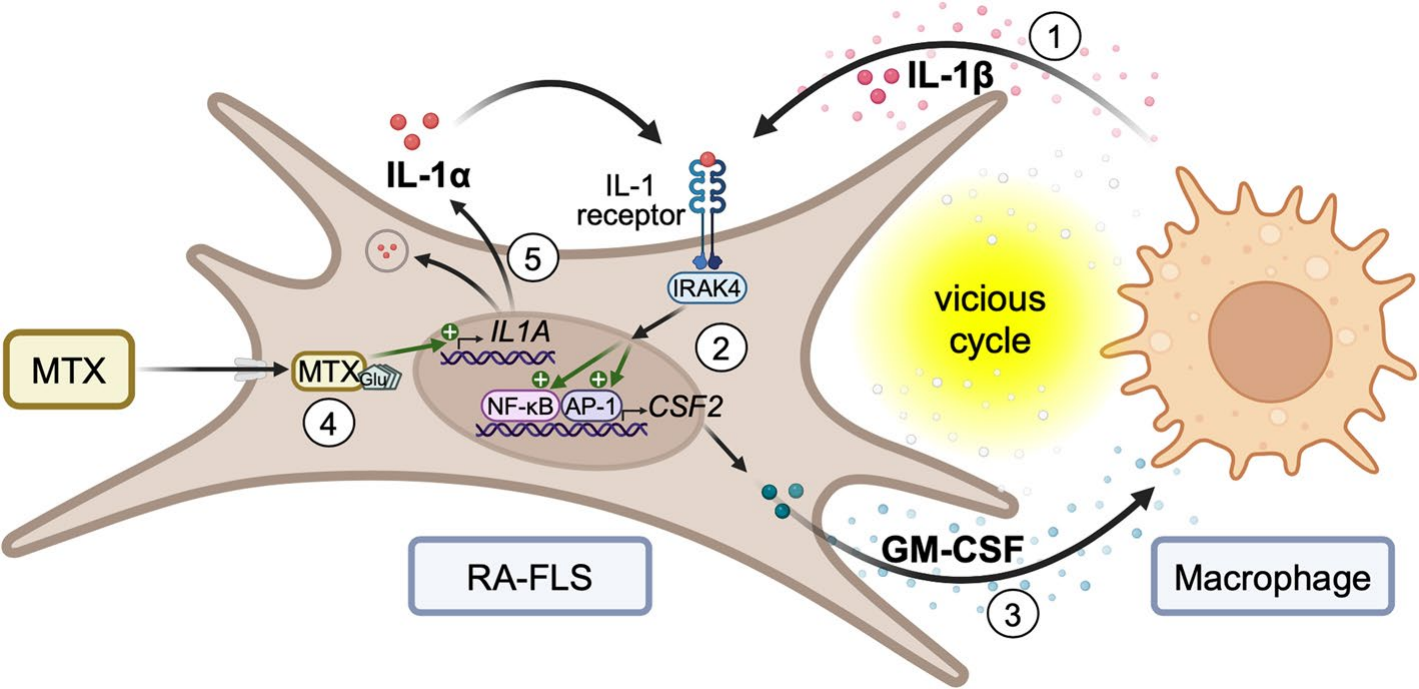
Proposed model for MTX-promoted production of GM-CSF in RA-FLS. (1) In the RA joint, cytokines like IL-1β secreted from macrophages activate FLS via IL-1 receptor signaling, inducing (2) a pro-inflammatory response including expression of *CSF2*/GM-CSF, via IRAK4 and the transcription factors NF-κB and AP-1. (3) GM-CSF released from FLS activates macrophages to produce IL-1β, forming a vicious cycle. (4) Treatment with MTX leads to accumulation of polyglutamated MTX in the cytosol of cells like FLS [60]. (5) MTX induces transcription of *IL1A*. By autocrine signaling, released IL-1α binds to the IL-1 receptor, thus promoting the vicious cycle. Illustration created with BioRender.com

Mechanistically, our results suggest that an increased release of IL-1α as a consequence of MTX treatment in activated RA-FLS promotes *CSF2*/GM-CSF expression in an autocrine fashion. This effect on *CSF2* expression could be abolished by selective inhibitors of IL-1 signaling (IRAK4 inhibitor and anakinra). Binding of IL-1 to its receptor triggers an intracellular signaling cascade that increases the expression of multiple pro-inflammatory genes [58]. We demonstrated that IL-1-induced *CSF2* expression in activated RA-FLS could be mediated via NF-κB or JNK pathways. The release of GM-CSF in turn activates macrophages to produce IL-1β, and MTX could thus enhance this vicious cycle. IL-1α is known to exist in both soluble and membrane-bound form and functions as a mediator of local inflammation [59]. It is constitutively expressed in cells of mesenchymal origin as a precursor (pro-IL-1α) which shuttles between the cytosol and the nucleus, and is biologically active if released from the cell. It is possible that cytosolic MTX causes secretion of preformed IL-1α. However, our data also demonstrate an increased transcription of *IL1A*, which may be mediated via earlier described DHFR inhibition and subsequent nitric oxide synthase uncoupling [60]. An overview of the proposed mechanisms and possible implications for the activation of RA-FLS and macrophages is illustrated in Fig. 6.

TOFA belongs to the novel JAK inhibitors, targeting signaling from type I (e.g. IL-6 and GM-CSF) and type II (e.g. interferon α, β and γ) cytokine receptors. A recent meta-analysis demonstrated the superior effect of combining TOFA and MTX as compared to MTX monotherapy [61], and JAK inhibition of GM-CSF effects on macrophages and neutrophils may be one mechanism. IL-1 signaling does not include JAK activation, nor does PDGF [21]. The most prominent effect of TOFA in our experimental set-up was inhibition of interferon-regulated gene expression, suggesting a significant autocrine signaling of interferon in IL-1 + PDGF-activated RA-FLS, as has been previously described with TNF-stimulated RA-FLS [23].

Data from the recent PEAC study strengthen the clinical relevance of both *CSF2*, *IL1B* and *IL1A* expression in the joint in early RA*.* Also, *CSF2* levels in the synovium could aid in predicting radiographic progression at 12 months [62]. Indeed, GM-CSF has been identified as an attractive novel target and several candidate drugs are under development for RA [63, 64]. The IL-1 receptor antagonist anakinra was approved in the early 2000s for treatment of DMARD-refractory moderate to severe RA [65]. MTX plus anakinra was demonstrated to be more effective than MTX monotherapy in large [66] and double-blinded [67] randomized controlled trials. However, anakinra is rarely used for RA in clinical practice today. Reasons for this include the development of more *clinically* effective DMARDs, the suboptimal pharmacokinetics of anakinra requiring daily subcutaneous injections, and the fact that a small open-label trial in early RA (CARDERA-2) demonstrated no *clinical* benefits of combining anakinra and MTX as compared to MTX in monotherapy [68]. Thus, it remains uncertain whether a specific RA patient population or subtype could benefit from therapies specifically targeting GM-CSF or IL-1. Our results showing that MTX promotes IL-1 signaling and thereby GM-CSF production, supports the combination of MTX with inhibitors targeting IL-1 signaling.

The proposed untoward effect of MTX could potentially contribute to persistence of the synovial inflammation in RA. However, it is difficult to predict how the experimental results translate into processes within the RA synovium. In the inflamed joint, the complex cytokine milieu and interactions with immune cells shape distinct RA-FLS states differing between the lining and sublining [69, 70]. Furthermore, Smith et al*.* found a strong IL-1β response signature particularly in activated lining FLS of RA synovium [70]. In two-dimensional culture, primary FLS undergo phenotypical changes influenced by the structural and molecular environment as well as passaging, e.g. leading to upregulation of the sublining marker CD90 [27, 69]. Consequently, conventional cultures do not optimally reflect the cell heterogeneity present in the synovium, and it remains to be elucidated how lining and sublining RA-FLS, respectively, respond to MTX in vivo. Moreover, the RA-FLS used in the experiments were derived from patients with end-stage RA subjected to different earlier treatments, which may have primed the cells to the variable responses observed in the experiments.

Optimally, synovial samples (synovial fluid or biopsies) collected from RA patients before and after MTX treatment could validate the in vitro findings and elucidate the implications of increased IL-1 and GM-CSF expression. Although blood samples are more routinely available, they may not reflect the changes taking place at the local site of inflammation. As demonstrated in the PEAC data, synovium gene expression was a stronger predictor of clinical response to DMARD treatment than blood [51]. Advancements in the use of synovial biopsies will provide a valuable tool for detailed understanding of RA pathogenesis and treatment response. Novel insights into the mechanisms of action as well as off-target effects of MTX could lead to improved treatment strategies and identification of response biomarkers.

## Conclusions

Unbiased transcriptomic analysis revealed unexpected, pro-inflammatory effects of MTX on activated primary RA-FLS. We demonstrate that therapeutic concentrations of MTX induce secretion of IL-1 from RA-FLS which augments their release of GM-CSF and activation of macrophages. This untoward effect of MTX might contribute to the persistence of synovitis and other disease manifestations and of co-morbidities.

## Supplementary Information

Additional file 1. Supplementary material.

## Data Availability

The RNA-seq datasets generated and analyzed in the current study were deposited into the Gene Expression Omnibus database, accession number GSE266310, available at https://www.ncbi.nlm.nih.gov/geo/query/acc.cgi?acc=GSE266310.

## Abbreviations

ANOVA: Analysis of variance
DAS28-ESR: Disease Activity Score 28-joint count with Erythrocyte Sedimentation Rate
CT: Cycle threshold
DHFR: Dihydrofolate reductase
DMARD: Disease-modifying antirheumatic drug
DMEM: Dulbecco’s modified Eagle’s medium
FBS: Fetal bovine serum
FLS: Fibroblast-like synoviocytes
GM-CSF: Granulocyte–macrophage colony-stimulating factor
IL: Interleukin
IRAK4: Interleukin-1 receptor-associated kinase 4
JAK: Janus kinase
JNK: Jun N-terminal kinase
M-CSF: Macrophage colony stimulating factor
mRNA: Messenger RNA
MTX: Methotrexate
NF-κB: Nuclear factor κB
PDGF: Platelet-derived growth factor
qPCR: Quantitative real-time PCR
RA: Rheumatoid arthritis
RNA-seq: RNA sequencing
SEM: Standard error of the mean
STAT: Signal transducer and activator of transcription
TOFA: Tofacitinib
