# Supplementary material for: Methotrexate promotes the release of granulocyte–macrophage colony-stimulating factor from rheumatoid arthritis fibroblast-like synoviocytes via autocrine interleukin-1 signaling
Source: Arthritis Res Ther. 2024 Oct 11;26:178. doi: 10.1186/s13075-024-03406-6 (PMC11468154; doi:10.1186/s13075-024-03406-6)
Supplement: Supplementary file 1 — Additional file 1. Supplementary material. [file 13075_2024_3406_MOESM1_ESM.pdf]

SUPPLEMENTARY MATERIAL

Methotrexate promotes the release of granulocyte-macrophage colony-stimulating factor from rheumatoid arthritis fibroblast-like synoviocytes via autocrine interleukin-1 signaling

Beatrice Bergström[1], Tilia Selldén[1], Miriam Bollmann[1,2], Mattias N.D. Svensson[1,2] and Anna-Karin Hultgård Ekwall[1,3*]

[1]Department of Rheumatology and Inflammation Research, Institute of Medicine, Sahlgrenska Academy, University of Gothenburg, Gothenburg, Sweden

[2]SciLifeLab, University of Gothenburg, Gothenburg, Sweden

[3]Department of Rheumatology, Division 3, Sahlgrenska University Hospital, Gothenburg, Sweden

Supplementary tables

Supplementary Table 1. Demographics and clinical data of a cohort of early RA patients.

	Early RA patients (*n* = 24)	
**Variable**	**Baseline**	**2-year follow-up**
**Age**, median, years (range)	56.5 (22-74)	
**Female sex**, n (%)	21 (88%)	
**Symptom duration at diagnosis**, median, months (range)	6 (1-31)	
**Erosive disease**, n (%)	5 (21%)	
**SJC (28)**, mean (± SD)	7.4 (± 4.0)	0.8 (± 1.2)
**TJC (28)**, mean (± SD)	6.5 (± 4.2)	1.9 (± 3.4)
**CRP**, mean (± SD)	18.3 (± 24.0)	4.1 (± 5.0)
**ESR**, mean (± SD)	27.6 (± 24.8)	16.7 (± 13.2)
**RF positive**, n (%)	20 (83%)	
**ACPA positive**, n (%)	19 (79%)	
**RF and ACPA positive**, n (%)	16 (67%)	
**DAS28**, mean (± SD)	4.8 (± 1.1)	2.7 (± 1.0)
**DAS28-CRP**, mean (± SD)	4.6 (± 1.0)	2.4 (± 0.9)
**CDAI**, mean (± SD)	22.6 (± 9.0)	5.9 (± 6.2)
**Therapy at 2 years**		
MTX monotherapy, n (%)		7 (29%)
MTX combination therapy[a], n (%)		11 (46%)
No MTX[b], n (%)		6 (25%)

[a] MTX combined with other bDMARD, csDMARD or prednisone

[b] Other DMARDs or prednisone

Supplementary Table 2. TaqMan Gene Expression Assays used for qPCR.

**Gene**		**Assay ID**
CDKN1A	Cyclin Dependent Kinase Inhibitor 1A	Hs00355782_m1
CSF2	Granulocyte-Macrophage Colony-Stimulating	Hs00929873_m1
GAPDH	Glyceraldehyde-3-Phosphate Dehydrogenase	Hs99999905_m1
IL1A	Interleukin 1 Alpha	Hs00174092_m1
IL1B	Interleukin 1 Beta	Hs01555410_m1
IL6	Interleukin 6	Hs00174131_m1
TGFA	Transforming Growth Factor Alpha	Hs00608187_m1

Supplementary Table 3. RA risk genes differentially expressed (adj. $p \leq 0.05$) in methotrexate-treated PDGF + IL-1β-activated RA-FLS compared to untreated control.

Gene symbol	Description
AFF3	ALF Transcription Elongation Factor 3
ARID5B	AT-Rich Interaction Domain 5B
B3GNT2	UDP-GlcNAc:BetaGal Beta-1,3-N-Acetylglucosaminyltransferase
BACH2	BTB Domain And CNC Homolog 2
C1QBP	Complement C1q Binding Protein
CD83	CD83 Molecule
CDK2	Cyclin Dependent Kinase 2
CDK6	Cyclin Dependent Kinase 6
CEP57	Centrosomal Protein 57
COG6	Component Of Oligomeric Golgi Complex 6
CSF2	Granulocyte-Macrophage Colony-Stimulating Factor
EOMES	Eomesodermin
ETS1	ETS Proto-Oncogene 1, Transcription Factor
ETV7	ETS Variant Transcription Factor 7
IFNGR2	Interferon Gamma Receptor 2
IL20RB	Interleukin 20 Receptor Subunit Beta
ILF3	Interleukin Enhancer Binding Factor 3
IRF4	Interferon Regulatory Factor 4
JAZF1	JAZF Zinc Finger 1
LBH	LBH Regulator Of WNT Signaling Pathway
NFKBIE	NFKB Inhibitor Epsilon
PLCL2	Phospholipase C Like 2
POU3F1	POU Class 3 Homeobox 1
PPIL4	Peptidylprolyl Isomerase Like 4
PRDM1	PR/SET Domain 1
PRKCH	Protein Kinase C Eta
PTPN22	Protein Tyrosine Phosphatase Non-Receptor Type 22
PVT1	Pvt1 Oncogene
RAD51B	RAD51 Paralog B
RCAN1	Regulator Of Calcineurin 1
REL	REL Proto-Oncogene, NF-KB Subunit
SPRED2	Sprouty Related EVH1 Domain Containing 2
STAT4	Signal Transducer And Activator Of Transcription 4
TNFAIP3	TNF Alpha Induced Protein 3
TNFRSF9	TNF Receptor Superfamily Member 9
TRAF1	TNF Receptor Associated Factor 1
WDFY4	WDFY Family Member 4

Supplementary Table 4. RA risk genes differentially expressed (adj. $p \leq 0.05$) in tofacitinib-treated PDGF + IL-1β-activated RA-FLS compared to untreated control.

Gene symbol	Description
ARID5B	AT-Rich Interaction Domain 5B
CDK6	Cyclin Dependent Kinase 6
ETV7	ETS Variant Transcription Factor 7
HLA-A	Major Histocompatibility Complex, Class I, A
HLA-B	Major Histocompatibility Complex, Class I, B
PRDM1	PR/SET Domain 1
RCAN1	Regulator Of Calcineurin 1
RUNX1	RUNX Family Transcription Factor 1
TLE3	TLE Family Member 3, Transcriptional Corepressor
TNFAIP3	TNF Alpha Induced Protein 3
TNFRSF9	TNF Receptor Superfamily Member 9

Supplementary figures

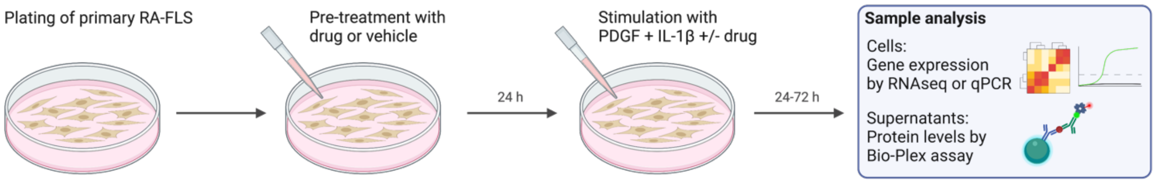

Supplementary Fig. 1 Overview of the experimental setup. Primary RA-FLS were plated, pre-treated with methotrexate or tofacitinib or vehicle for 24 h, followed by stimulation with PDGF-BB and IL-1β in the presence or absence of the drug for 24-72 h prior to analysis. Illustration created with BioRender.com.

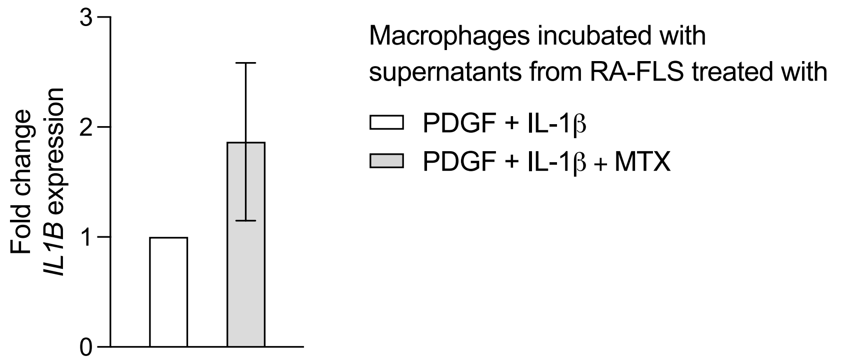

Supplementary Fig. 2 Expression of *IL1B* in macrophages incubated with supernatants from MTX-treated activated RA-FLS. Monocytes were isolated from buffy coats from healthy blood donors, plated and differentiated into macrophages. In parallel, RA-FLS were pre-treated with MTX or vehicle for 24 h followed by stimulation with PDGF + IL-1β in the presence or absence of MTX for 6 h. Media were then removed and the RA-FLS were incubated with fresh 1% FBS media for 72 h. Thereafter, the supernatants were collected and transferred to the macrophages. After 24 h incubation, macrophages were subjected to RNA isolation and analysis of *IL1B* expression by qPCR. Bar graph shows mean ± SEM (n = 3 different RA-FLS lines).

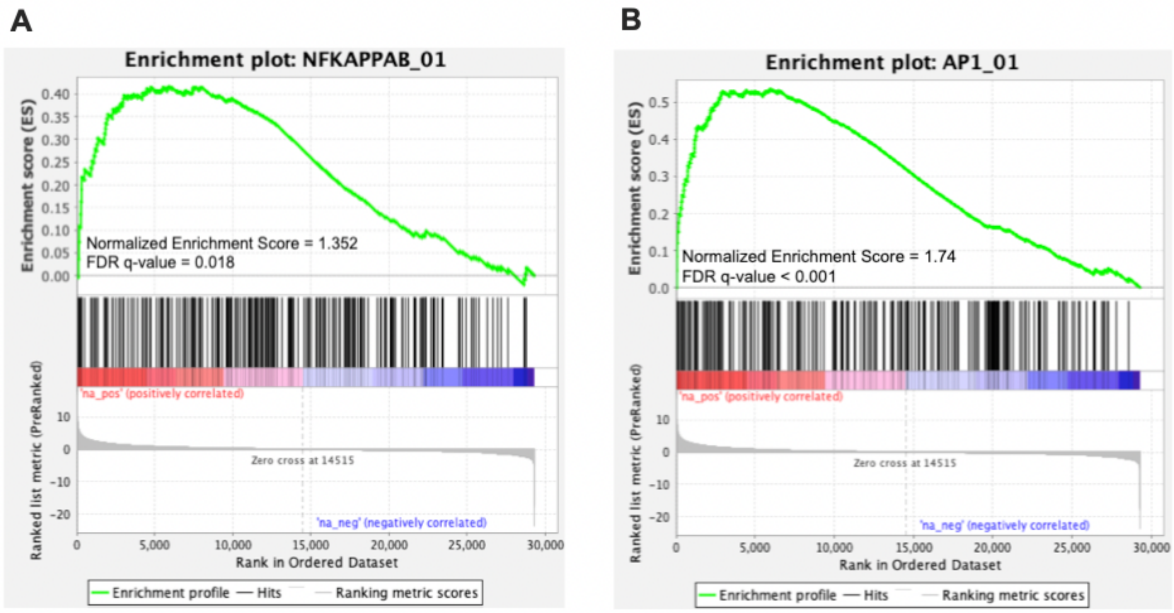

Supplementary Fig. 3 Gene Set Enrichment Analysis (GSEA) for **A** NF-κB transcription factor target genes/'NFKAPPAB_01' gene set (MSigDB) or **B** AP-1 transcription factor targes genes/'AP1_01' gene set (MSigDB), ran against a log_2 fold change-ranked list of genes from the RNA-seq expression dataset of MTX-treated versus untreated activated RA-FLS.

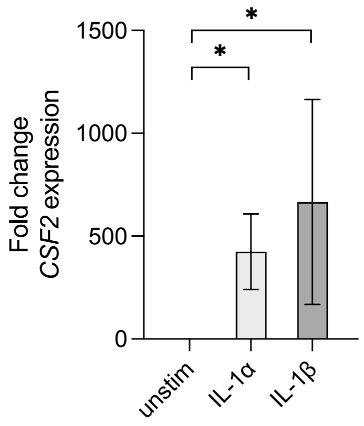

Supplementary Fig. 4 Effects of IL-1α and IL-1β on *CSF2* expression in RA-FLS. Primary RA-FLS were serum-starved overnight (in 1% FBS-DMEM), then stimulated with IL-1α or IL-β (both at 2 ng/mL) for 24 h prior to analysis of *CSF2* expression by qPCR. Bar graphs show mean ± SEM (n = 3 different RA-FLS lines). *$p < 0.05$ by one-way repeated measures ANOVA with multiple comparisons.

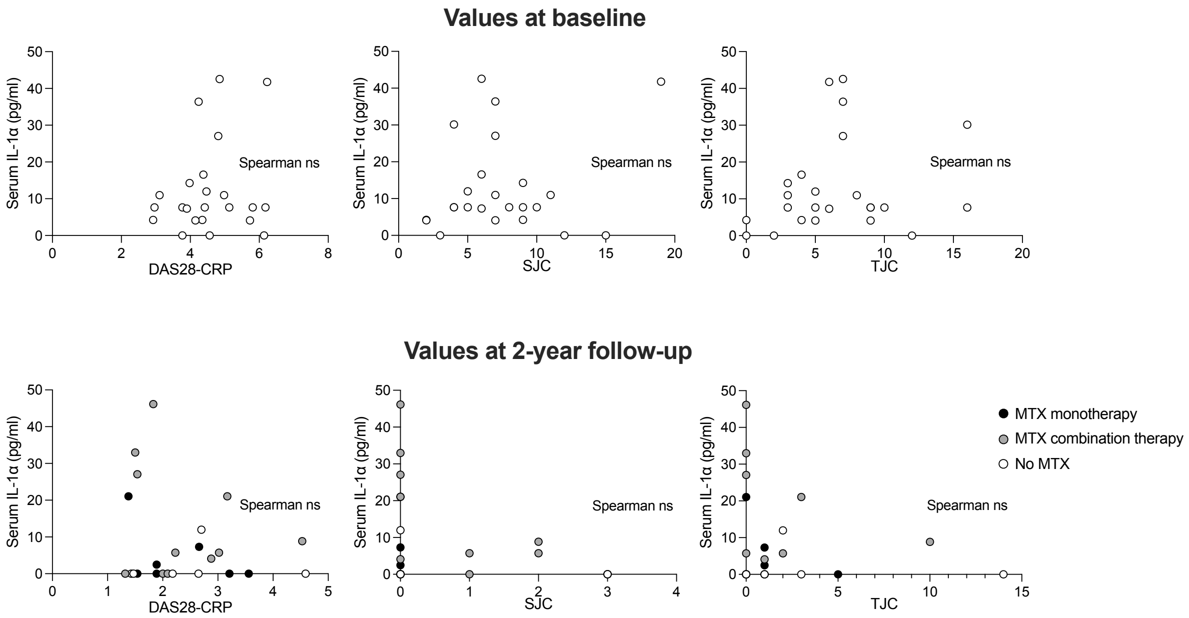

Supplementary Fig. 5 Serum levels of IL-1α in relation to disease activity marker DAS28-CRP, swollen joint count (SJC), or tender joint count (TJC) in a cohort of early RA patients at baseline and after two years of treatment as indicated.
